# Directing memory content to attentional templates: The finiteness effect of predictive information

**DOI:** 10.3389/fpsyg.2022.1082437

**Published:** 2023-01-06

**Authors:** Zhen Chen, Qiankai Li, Xinyu Li

**Affiliations:** ^1^Department of Psychology, Zhejiang Normal University, Jinhua, China; ^2^Key Laboratory of Intelligent Education Technology and Application of Zhejiang Province, Zhejiang Normal University, Jinhua, China

**Keywords:** attention, working memory, predictive cue, short term memory, template

## Abstract

Visual search can be accelerated according to the properties of information stored in memory and prior knowledge of the upcoming work. This helps the searcher direct their attention to (or avoid) items that match these properties. Meanwhile, different functional areas where these properties exist become attentional templates. Compared with neutral conditions, the use of attentional templates significantly benefits reaction time (RT). However, previous studies might have confounded the memory-driven and cue-driven effects. Thus, it is less clear which factor influences the template benefits. Modeled on previous research, this study employed a new design to explore the independent effects of textual cues, thus finding an inverse effect. More specifically, positively cueing an item retained in memory did not improve behavioral performance, whereas negatively cueing an item did achieve such an enhancement. Moreover, positive cueing even resulted in some damage to attentional searching under some conditions, thus indicating that the advantages of positive cueing reported in previous studies may be driven by working memory, while the effects of negative cueing are driven by prior knowledge.

## Introduction

Storing a specific feature of upcoming work before searching for its related target is an effective way to interact with the environment. Known as a ‘target template’, this type of information is maintained in the visual working memory (VWM). Thus, a subsequent attention search is biased toward representations in the list matching the target information (Duncan and Humphreys, 1989; Desimone and Duncan, 1995). To test how these template representations guide attention, most studies have employed the dual-task paradigm, in which before the relevant target can be detected from the list of distractors and the observer must remember a feature. In this context, many studies have found that if the cue matches the target when compared to a neutral baseline where the cue contains no information search, the performance will improve (Wolfe et al., 2004; Vickery et al., 2005; Töllner et al., 2010). In neurophysiology, sustained activity in the lateral parieto-occipital regions was found when subjects stored target-related representations during the delay period (Chelazzi et al., 1993; Carlisle et al., 2011), thereby indicating that such working memory content influences the upcoming search toward its related objects.

Attentional guidance can also be performed by inhibiting distractors as a supplement to facilitate visual search; more precisely, any distractor objects in the array are removed from the scanning process (Gaspelin and Luck, 2018). These results indicate that by excluding target irrelevant items from an attentional array, non-target information can also be used effectively to facilitate visual search in predictive environments. Recent evidence has shown that such attentional suppression is implemented by reducing the weights of distractor (no-target) features and not increasing the weights of target features (Moher et al., 2014; Nie et al., 2016). Furthermore, the creation of a negative (distractor) attentional template, which dictates information to be avoided, may constitute an underlying mechanism for distractor suppression in search guidance (Duncan and Humphreys, 1989; Humphreys and Müller, 1993). Compared with the positive (target) template, the negative template attenuated the activation of features associated with the distractors labeled as avoidance, thus reducing their potential for competitive selection. Hence, both positive (target) and negative (distractor) templates support the prediction of task-related goals in the generative environment (see Conci et al., 2012).

Both distractor suppression and target selection appear to modulate searches in predictive environments. Previous studies on setting negative (interference) or positive (target) cues before searching the array have shown that priming a set of non-target or target features in the upcoming search array improves task performance; the observer can set negative or positive templates to suppress unrelated items and accelerate the goal items, respectively. These studies have typically employed a condition-blocked design, in which one block contains one textual cue type (positive/negative) and prior knowledge (textual cue) is presented before each block starts. Thus, subjects know that the subsequent memory item is a distractor or target before a block starts, and accordingly, this prior information helps them speed up the attentional task (Arita et al., 2012; Kugler et al., 2015; Reeder et al., 2017; Conci et al., 2019).

Of note, most studies have reported that negative distractor cues tend to produce smaller benefits in comparison with positive target cues, thereby demonstrating that negative templates are relatively more difficult to utilize and that prior knowledge of the upcoming task plays a more important role in target selection than distractor suppression (Kugler et al., 2015). In this study, each trial of the cueing condition involved a memory item with predictive information and a memory-target-matched/non-target-matched visual search test, including a search for an unrelated color that was presented to be remembered in the neutral condition. However, such an approach may cause confusion because other studies have reported that representations are maintained in the memory automatic bias attention direction when searching for memory content-matched items (Anderson et al., 1997; Logan and Gordon, 2001; Soto et al., 2008). Thus, memory items and predictive cues produce a top-down effect (automatic guidance vs. search strategy), relative to the neutral condition. The results of these experiments may confound the memory-driven and predictive cue-driven effects rather than demonstrate the effects of the predictive cue itself.

To clarify this issue and further examine the role of VWM representations in cueing effect, we conducted two experiments in reference to the study conducted by Arita et al. (2012). In Experiment 1, we replaced the neutral condition with memory-item-target-match (MM) and memory-item-non-target-match (MN) conditions and then set the same color of stimulants in a fixed region (Arita et al., 2012; Exp. 1, 2, 3). To exclude the effect of the search strategy, we randomly altered the colors of the search display in Experiment 2. Moreover, in the present study, we mixed the conditions in a block and the textual cue (prior knowledge) about the WM content was set in each trial (Beck et al., 2018).

In sum, we found that when controlling for memory representation as an additional variable, the negative cue produced more benefits than the positive cue. Moreover, this effect remained even after increasing the search load and controlling the strategy. These results are completely contrary to those found in previous studies (Kugler et al., 2015). Surprisingly, the positive cue even caused some damage to the subsequent visual search. This implies that tactic cues play different roles in each of the two inversed channels that facilitate the transformation of initial memory contents into different attentional templates according to top-down predictive cues.

## Materials and methods

### Experiment 1

Experiment 1 employed a variant of the paradigm provided by Arita et al. (2012), with four implemented conditions, namely the memory-item-target-match (MM), memory-item-non-target-match (MN), cue-memory-item-target-match (C-MM), and cue-memory-item-non-target-match (C-MN). Here, we focused on any differences between the positive cue (target color) reaction time (RT) benefits (MM-CMM) and negative cue (non-target color) RT benefits (MN-CMN).

#### Experiment 1 methods

##### Participants

We recruited a total of 24 undergraduate students (3 males, 21 females; M age = 19.96; SD =1.16) from Zhejiang Normal University in China. For study inclusion, these participants were required to have normal or corrected-to-normal color vision and sufficient visual acuity. None withdrew from the experiment due to physical discomfort or subjective reasons. All data were included in the analysis. We determined the set size (n = 24) based on Arita et al. (2012), wherein the effect size (η^2^ = 0.23) indicated that at least 18 participants were required to achieve 80% power.

Each participant provided informed consent, per the Declaration of Helsinki. The research plan was approved by the Behavioral and Social Sciences Institutional Review Board of Zhejiang Normal University. Each participant was given monetary compensation (30 RMB) after completing the experiment.

##### Visual stimuli and apparatus

We controlled the stimuli and response registration using Python scripts.^1^ Participants were tested in a dim room with their chins positioned on a chinrest located 70 cm from a 17-in LCD monitor (resolution: 1024 ⫻ 768; refresh rate: 85 Hz).

**Figure 1:**
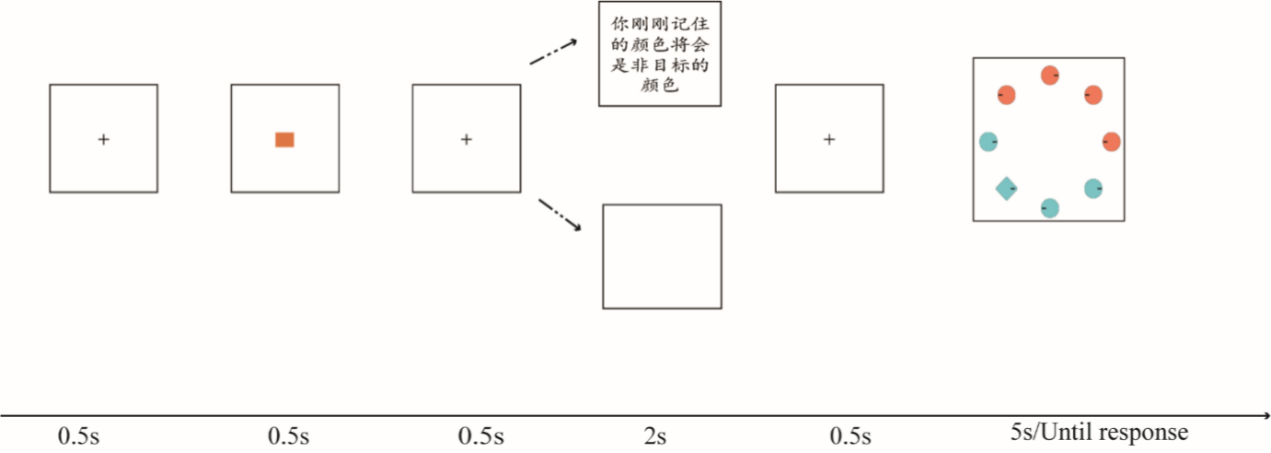
Experiment 1 task. The first portions of all trials were identical, beginning with a fixation dot (0.5 s), followed by a memory item (0.5 s), then a predictive cue (2 s) presented to indicate the fate of the remembered item (target or non-target); next, a search test was presented until the response. Participants were required to search for a diamond in a set of circles, then make a judgment about the location of the line. In the no-cue condition, the remembered color was required to be presented in the search display as a target feature or distractor feature. The predictive cues were 100% valid.

After remembering a color at the beginning of the search, 4/8/12 colored shapes (1° × 1°) were presented on an imaginary circle with a radius of 4° at the same angle deviation and centered at the fixation, against a gray background (Figure 1). The shapes were of two forms, namely 3/7/11 circles and one diamond, whereas two stimuli colors were randomly selected from 12 color values. The colors were selected from a set of 180 color values evenly distributed along with a color wheel in L*a*b* (L* = 70, a* = 20, b* = 38) color space at intervals of 30°.

##### Procedure

We used a 2*2*3 repeated-measures design with predictive cue type: cue/no cue, match type: target match/non-target match, and set size: 4/8/12 as three factors. Thus, each set size included four types of test conditions: cue-target match (positive cue), no-cue-target match, cue non-target match (negative cue), and no-cue non-target match. We set three blocks and each contained one search load of the mixed conditions. The order of conditions was also counterbalanced across the participants. Experiment 1 began with 20 practice trials, following the instructions regarding the cue color and its relationship to the target color.

The participants performed 420 trials. Each trial began with a black fixation at the center of the screen for 0.5 s, followed by a color they needed to remember for 0.5 s, also placed at the center of the screen. The remembered color had two conditions: target match or non-target match. At this time, one of three possible arrangements was presented at the center of the screen for 2 s, including two predictive cues written in Chinese: (1) “the color you have just remembered will be the target color”” (target match cue/positive cue); (2) “the color you have just remembered will be the non-target color” (non-target match cue/negative cue); and (3) empty screen. Finally, the participants were presented with a visual search task containing 4/8/12 items, wherein they were prompted to locate a diamond from a set of circles and then indicate the location of the embedded line (i.e., right or left). As in Arita et al. (2012), only two colors were presented in the search task, with half displayed in the color to be remembered, and all items always symmetrized in a fixed region of the screen. In each condition, the location of the target was pseudorandomized (Figure 1).

#### Experiment 1 results

The accuracy of each condition was on the ceiling (>98%); thus, we did not include it in the analysis. We excluded wrong response trials from the analysis and conducted a repeated-measures analysis of variance (ANOVA) on the RTs and related benefits. The ANOVA on RTs revealed a significant main effect for cue type [*F* (1,23) = 30.15, *p* < 0.001, *η^2^_p_* = 0.57], match type [*F* (1,23) = 96.16, *p* < 0.001, *η^2^_p_* = 0.81], and set size [*F* (2,46) =16.15, *p* < 0.001, *η^2^_p_* = 0.41]. Further comparisons indicated that no-cued trials responses were longer than cued trials [*t*(23) = −5.49, *p* < 0.001, *Cohens’d* = −1.12]; target match trials responses outperformed non-target match trials [*t*(23) = −9.81, *p* < 0.001, *Cohens’d* = −2]; and set size 4 showed faster RT than set size 8 [*t*(23) = −4.38, *p* < 0.001, *Cohens’d* = −0.89] and set size 12 [*t*(23) = −5.33, *p* < 0.001, *Cohens’d* = −1.09].

**Figure 2:**
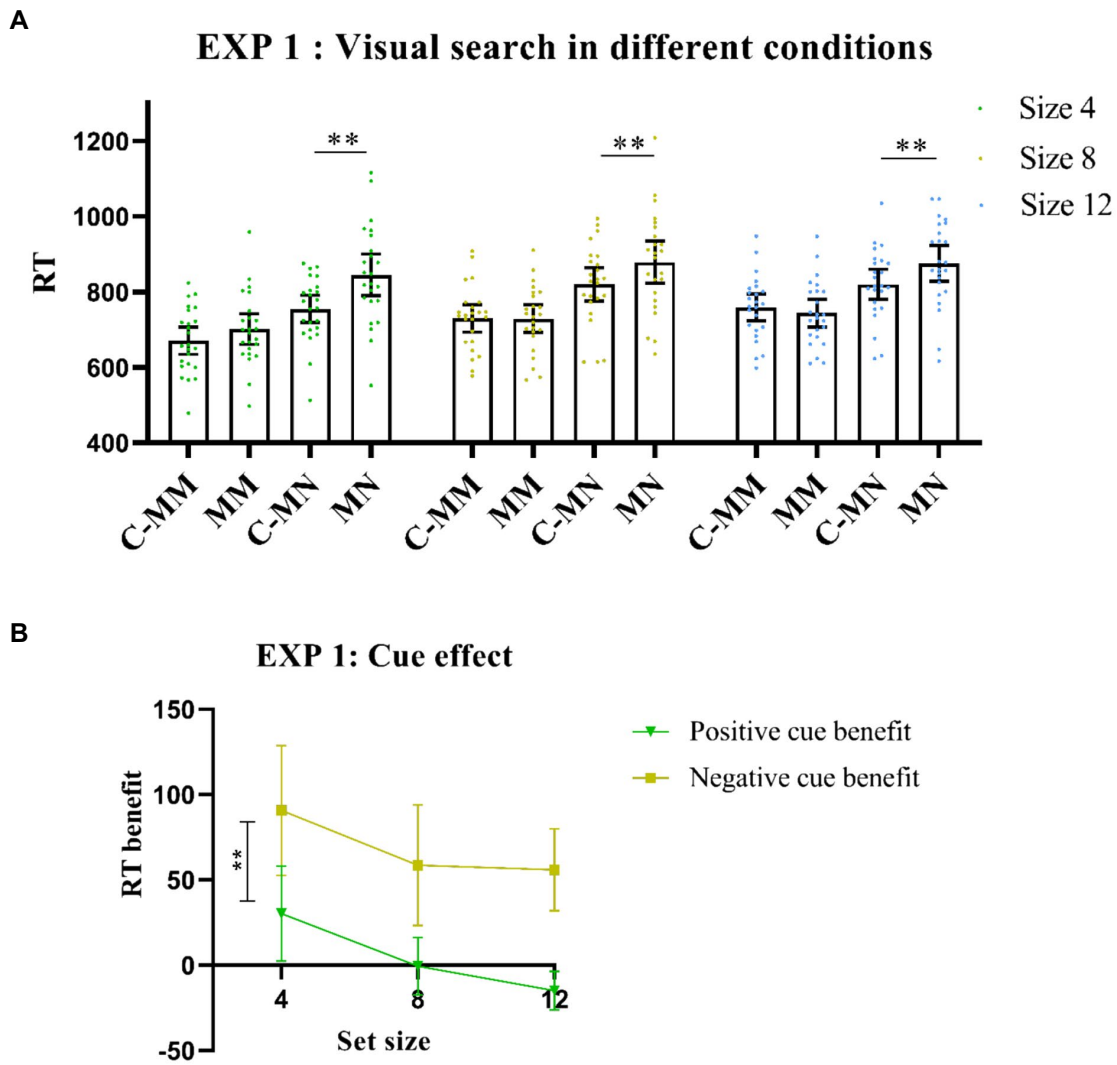
**(A)** Visual search performance in all Experiment 1 conditions. MM: memory-item-target-match; MN: memory-item-non-target-match; C-MM: cue-memory-item-target-match; CMN: cue-memory-item-non-target-match. **p < 0.001. Error bars represent 95% within-subjects confidence intervals, as described by Loftus and Loftus (1988). **(B)** Cue benefit in Experiment 1. **p < 0.001. Error bars represent 95% within-subjects confidence intervals, as described by Loftus and Loftus (1988). Positive cue benefit = MM-CMM(RT); Negative cue benefit = MN-CMN(RT).

Importantly, there was also a significant interaction between cue type and match type [*F* (1,23) = 25.12, *p* < 0.001, *η^2^_p_* = 0.52], with a longer RT of non-target match trials under no-cue condition than under cue condition [*t*(23) = −7.43, *p* < 0.001]. However, RT values of target-matching tests were similar between the cue and no-cue conditions [*t*(23) = −0.53, *p*>0.05]. The results indicated that the effect of the cue was much larger for the non-target match (negative) than for the target match (positive). The interactions between set size and cue type were also significant [*F* (2,46) = 4.84, *p* < 0.05, *η^2^_p_* = 0.17]. However, match type and set size were not significant [*F* (2,46) = 2.02, *p*> 0.05, *η^2^_p_* = 0.08], nor was the three-way interaction [*F*(2,46) = 0.24, *p* >0.05, *η^2^_p_* = 0.01; Figure 2A].

We also calculated RT benefits for the positive and negative cues and then stored this as a new analysis variable. Next, we conducted a 2*3 ANOVA with RT benefits and set size as the two levels. The statistical results showed a main effect for cue benefit [*F* (1,23) = 25.12, *p* < 0.001, η^2^_p_ = 0.52], whereas the *post-hoc* test showed that the negative cue benefit was significantly higher than the positive cue benefit [*t* (23) = −5.01, *p* < 0.001, *Cohens’d* = −1.02; Figure 2B].

In Experiment 1, the negative textual cue thus produced more benefits than the positive cue. While prior knowledge of the distracting color enhanced performance, knowledge of the upcoming target color did not always lead to performance benefits.

#### Experiment 1 discussion

Participants responded better under positive (vs. negative) cues, which supports previous findings. However, the cueing effect of the positive cue failed to be observed in Experiment 1. This implies that the positive cue may not produce substantial benefits in the context of informing observers about the color of an upcoming visual search target. Meanwhile, the negative cue produced relative benefits. Taken together, these results provide evidence that the negative cue benefit is larger than the target cue benefit.

### Experiment 2

In Experiment 1, we examined the effects of predictive cues after excluding the influence of the memory item. Our results were directly opposite to those reported in previous studies. However, our color symmetry settings might have led to a potential problem, as participants could have strategically used that information when giving their responses. In Experiment 2, we eliminated this possibility by randomly mixing colors in the search array.

#### Experiment 2 methods

While the Experiment 2 apparatus, stimuli, design, and procedure were similar to those in Experiment 1, we implemented some important changes. Specifically, we randomly shuffled colors’ locations in the visual search display and increased the trial number to 560 across three blocks. The participants included 24 individuals who did not participate in Experiment 1 (four males, 20 females; M age = 20.75; SD = 1.91). All had normal or corrected-to-normal vision and were right-handed. All other details were the same as in Experiment 1.

#### Experiment 2 results

For the same reason, accuracy was not included in the statistical analysis in experiment 2. The ANOVA on RTs revealed a significant main effect for cue type [*F* (1,23) = 7.36, *p* < 0.05, *η^2^_p_* = 0.24], match type [*F* (1,23) = 95.65, *p* < 0.001, *η^2^_p_* = 0.81], and set size [*F* (2,46) = 34.19, *p* < 0.001, *η^2^_p_* = 0.60]. The comparisons of main effects indicated that no-cued trials responses took longer than cued trials [*t*(23) = −2.71, *p* < 0.05, *Cohens’d* = − 0.55] and target match trials responses outperformed those of target un-match trials [*t*(23) = −9.78, *p* < 0.001, *Cohens’d* = −2].

**Figure 3:**
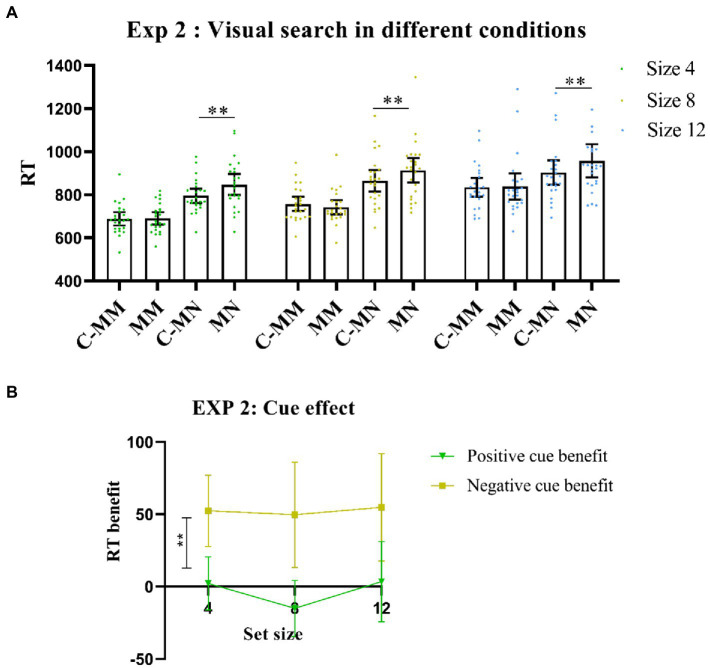
**(A)** Visual search performance in all Experiment 2 conditions. MM: memory-item-target-match; MN: memory-item-non-target-match; C-MM: cue-memory-item-target-match; CMN: cue-memory-item-non-target-match. **p < 0.001. Error bars represent 95% within-subjects confidence intervals, as described by Loftus and Loftus (1988). **(B)** Cue benefit in Experiment 2. **p < 0.001. Error bars represent 95% within-subjects confidence intervals, as described by Loftus and Loftus (1988). Positive cue benefit = MM-CMM (RT); Negative cue benefit = MN-CMN (RT).

Notably, there was also a significant interaction between cue type and match type [*F* (1,23) = 31.9, *p* < 0.001, *η^2^_p_* = 0.58], with a longer RT of non-target match trials under no-cue condition than under cue condition [*t*(23) = −5.08, *p* < 0.001]. Consistent with Experiment 1, RT values of target-matching tests were similar between the cue and no-cue conditions [*t*(23) = −0.31, *p* >0.05]. The interactions between set size and cue type were also significant [*F* (2,46) = 0.61, *p*> 0.05, *η^2^_p_* = 0.01]. However, match type and set size were significant [*F* (2,46) = 11.41, *p* < 0.001, *η^2^_p_* = 0.33], nor was the three-way interaction [*F* (2,46) = 0.3, *p* > 0.05, *η^2^_p_* = 0.01]. The results showed that predictive cues had a great benefit in the substance visual search task (Figure 3A).

We also calculated the RT benefits. Here, the ANOVA showed a significant main effect for cue benefit [*F* (1,23) = 31.90, *p* < 0.001, *η^2^_p_* = 0.58], whereas the *post-hoc* test showed that the negative cue benefit was significantly higher than the positive cue benefit [*t* (23) = −5.65, *p* < 0.001, *Cohens’d* = −1.15; Figure 3B].

#### Experiment 2 discussion

The mixed color presentation did not influence our results in Experiment 2. This further confirmed that the cueing effect of prior knowledge is limited in distractor suppression rather than in target selection. This finding was also consistent with our initial predictions, thus confirming that the construction of a different template may have independent and distinct components.

## General discussion

In this study, we conducted two behavioral experiments to retest the influences of two types of predictive cues (positive and negative), finding directly opposite results from those reported in previous studies (Wolfe et al., 2004; Vickery et al., 2005; Töllner et al., 2010; Kugler et al., 2015). Based on the methodology provided by Arita et al. (2012), Experiment 1 increased two conditions to manipulate the VWM matching effect. Here, the positive cue benefit was significantly smaller than the negative cue benefit and close to the baseline. Subsequently, Experiment 2 employed the same framework as Experiment 1, but with shuffled color symmetry. These results reinforced our initial conclusion. We also increased the set size to explore whether the observed effect was a function of attention load and confirm that the negative cue effect was stable. We believe that these results represent different components of the target and distractor templates.

To date, previous studies have produced mixed results; some have supported (Arita et al., 2012; Cunningham and Egeth, 2016; Reeder et al., 2017) and some have rejected (Beck and Hollingworth, 2015; Becker et al., 2016; Beck et al., 2018) the notion distractor inhibitory template. Our results support the basic findings reported by Arita et al. (2012) and thus provide new evidence reinforcing the existence of the template. As described in the introduction section, the completion of a visual search task after remembering an unrelated color may not work the same as at the baseline when compared to the positive or negative cue condition. At least two factors are involved in such an experiment, including (1) the relationship between the memory color and search color and (2) the predictive cue. The former represents an automatic driver linkage between memory and attention (Bundesen, 1990; Anderson et al., 1997; Logan and Gordon, 2001), whereas the latter represents a valid external strategy that modulates resource allocation (Wolfe et al., 2004; Vickery et al., 2005). These two completely different factors might have influenced the conclusions made in previous work.

The relatively small positive cue benefits can be attributed to a conflict between two distinct guiding factors. While we cannot currently provide sufficient evidence to prove this, we did find that the positive cue imposed significant damage to visual attention in some cases. Here, two top-down benefit strategies possibly competed for resources, as some researchers have reported that the representation obtaining the status of attentional template, whether at encoding or during maintenance, competes for the amount of WM resources proportional to its relevance for visual search (Huynh Cong and Kerzel, 2021).

Consistent with previous studies, we also found a target selection advantage (Arita et al., 2012; Cunningham and Egeth, 2016; Reeder et al., 2017). Distractor suppression consists of at least two procedural stages: (1) selecting and inhabiting the distractor feature and then (2) searching for the target (Chang and Egeth, 2019). Thereafter, target selection can directly search for the target using the former information. The RT differences may be because of the additional stage. Given that the larger RT benefits in the positive template observed in other studies cannot affect the trend of the RT differences (Arita et al., 2012; Kugler et al., 2015), our results also show that the target template maintains a priority effect in visual searches. However, the problem is determining the source of the target template advantage; our results indicate that it is derived through the relationship between WM and the target, including implicit knowledge about the future attention task. In contrast, if WM contents mismatch the future task and participants are given prior information, then this may reshape WM representations to adapt to the environment, thus generating better benefits than the mismatch condition.

Based on the separation of the two templates, our research provides evidence that the positive template can be accommodated by a “memory-driven” model, positing that perceptual attention is biased toward features maintained in the working memory (Desimone and Duncan, 1995). More precisely, the binding between working memory content and visual attentional search is strong and automatic (Woodman and Luck, 2007). If prior knowledge matches the memory content, the advantage of working memory matching will cover the slight advantage of prior knowledge, leading to no significant difference of predictive cue in the target-match condition in the present results. Meanwhile, the negative template is best suited to a ‘feature-based visual search’ model (Treisman and Gelade, 1980; Wolfe et al., 1989; Wolfe, 1994; Wolfe and Gray, 2007), which posits that top-down information supports the generation of a stronger weight for the coding of target (distractor) related features. More specifically, top-down task goals drive the direction of our attention; in the present study, the informative cues successfully improved the task performance in the non-target match condition, indicating the colored feature was directed to the visual search task actively.

There is still no clear answer whether the internal modes of different templates rely on shared neural mechanisms (Cowan, 1999; Yamaguchi et al., 2004) or if they are unique and operate at different levels of cognitive or neural processing (Reeder et al., 2017, 2018). While WM may potentially work as a source of such functional areas, it is not the only factor because learning also plays a critical role in such psychological activities (Geng et al., 2019). Even for the representations held in WM space, each may have its specific characteristics (Oberauer, 2002). Besides, there are some limitations concerning the results of behavioral measurements. Thus, future studies can explore the differences in the mechanism of different attention templates from the perspective of neurophysiology. Moreover, although the set size was increased to 12, the search task seemed easy and static in our experiment. This rendering mode reduces its similarity to real-dynamic scene search. These questions should be addressed in future studies.

The idea that we can configure our attention to select certain objects is attractive. However, while our findings demonstrate that we can use prior information to avoid specific features, this information cannot be used when memory items have already been matched to the target. We also found evidence suggesting that the cueing effects observed for target templates in previous studies might have been memory-driven, while the negative cueing effects were due to prior useful information. This indicates that the two attention templates involve completely different mechanisms.

## Data availability statement

The original contributions presented in the study are included in the article/supplementary material, further inquiries can be directed to the corresponding author.

## Ethics statement

The studies involving human participants were reviewed and approved by the Behavioral and Social Sciences Institutional Review Board of Zhejiang Normal University. The patients/participants provided their written informed consent to participate in this study.

## Author contributions

All authors listed have made a substantial, direct, and intellectual contribution to the work and approved it for publication.

## Funding

This research was supported by the Natural Science Foundation of Zhejiang Province (LY18C090007) to XL.

## Conflict of interest

The authors declare that the research was conducted in the absence of any commercial or financial relationships that could be construed as a potential conflict of interest.

## Publisher’s note

All claims expressed in this article are solely those of the authors and do not necessarily represent those of their affiliated organizations, or those of the publisher, the editors and the reviewers. Any product that may be evaluated in this article, or claim that may be made by its manufacturer, is not guaranteed or endorsed by the publisher.

## Footnotes

^1^ https://www.python.org
